# Thermophilic bio-factory *Bacillus sonorensis* SS1 for superparamagnetic iron sulfide nanoparticles (FeSNPs) synthesis: in vitro and molecular modeling studies of anticancer activity

**DOI:** 10.1186/s12866-026-05140-2

**Published:** 2026-07-16

**Authors:** Samia S. Abouelkheir, Shaimaa Makled, Botros Y. Beshay, Ahmed Abdel-Mawgood, Sarah O. Makled

**Affiliations:** 1 National Institute of Oceanography and Fisheries (NIOF), Cairo, Egypt; 2 Department of Pharmaceutics, Faculty of Pharmacy, Alexandria University, Alexandria, Egypt; 3 Pharmaceutical Chemistry Department (Pharmaceutical Sciences Division), College of Pharmacy, Arab Academy for Science, Technology and Maritime Transport, Alexandria, Egypt; 4 Biotechnology Program, Faculty of Basic and Applied Sciences, University of Science and Technology, Alexandria, 21934 Egypt; 5 Oceanography Department, Faculty of Science, Alexandria University, Alexandria, Egypt

**Keywords:** Cytotoxicity, FeSNPs, Fish pathogen, *Bacillus sonorensis* SS1, Thermophilic, Brunauer-Emmett-Teller (BET)

## Abstract

An eco-friendly method lets the thermophilic *Bacillus sonorensis* SS1 synthesize superparamagnetic nano-sized iron sulfide particles (FeSNPs) as bio-factories. These clean and non-toxic FeSNPs have led to increased interest in using inorganic nanoparticles (NPs) in health materials and industrial products. The biosynthesized FeSNPs exhibited an average particle size of approximately 4.5 nm and a negative surface charge (− 16.8 ± 0.6 mV), indicating good colloidal stability. In vitro cytotoxicity evaluation using the MTT assay demonstrated concentration-dependent growth inhibition against five cancer cell lines (A549, HeLa, MCF-7, HepG2, and HCT-116), with IC₅₀ values ranging from 18 to > 100 µg/mL. The highest cytotoxic activity was observed against A549 (IC₅₀ = 18 µg/mL) and HeLa (IC₅₀ = 27 µg/mL) cells, and MCF-7 (IC₅₀ = 75 µg/mL) showed moderate sensitivity, while comparatively lower sensitivity was detected in HepG2 and HCT-116 cells (IC₅₀ value > 100 µg/mL). In contrast, normal peripheral blood mononuclear cells (PBMCs) exhibited minimal cytotoxicity, with an IC₅₀ value > 100 µg/mL, indicating selective anticancer activity of the FeSNPs. The molecular docking studies demonstrated that the FeSNPs exhibit a strong binding affinity to the active sites of Caspase-3, VEGFR, and Aurora-A. These interactions suggest that FeSNPs can effectively inhibit critical pathways involved in tumor growth, angiogenesis, and radiotherapy resistance. The biochemical validation confirmed these interactions, showing a 14.6-fold induction of active Caspase-3 (516.8 ± 20.1 pg/mL) in A549 cells. Additionally, FeSNPs exhibited potent inhibitory activity against VEGFR2 (IC₅₀ = 0.447 ± 0.019 µg/mL) and Aurora-A (IC₅₀ = 0.268 ± 0.012 µg/mL). These findings demonstrate the potential of FeSNPs as multifunctional agents capable of simultaneously triggering apoptosis and disrupting angiogenic and proliferative pathways. The findings focus on the potential of FeSNPs as versatile therapeutic agents in cancer therapy.

**Graphical Abstract:**

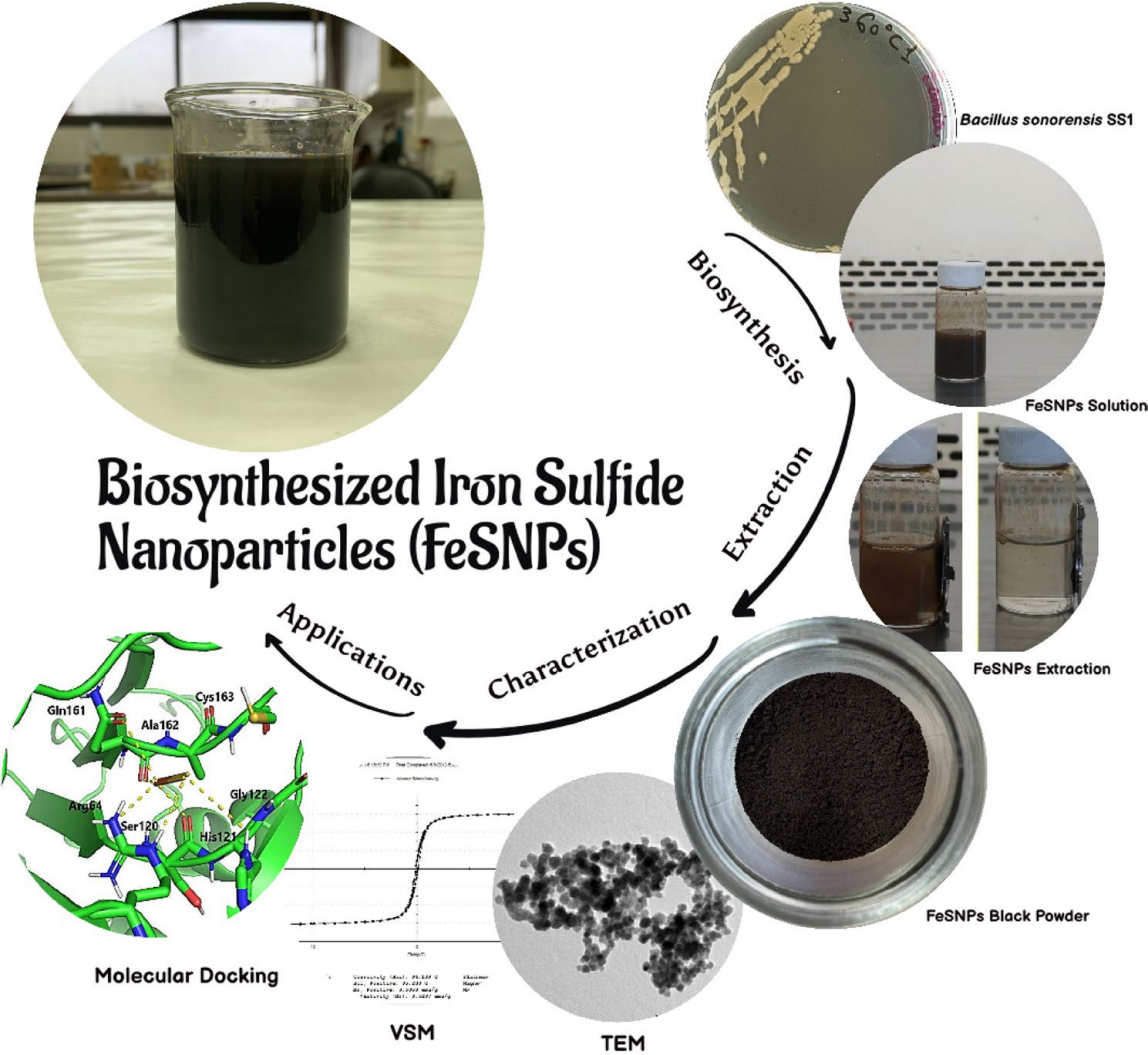

## Introduction

Nanotechnology has revolutionized the advancement of therapeutic medicines and technologies [1, 2], coupled with iron-based nanomaterials being a popular choice for biomedical applications such as drug delivery, magnetic resonance imaging (MRI), bioseparation, biosensors, and tumor hyperthermia [3]. These nanomaterials are a new generation of artificial enzymes because they possess features that are similar to those of enzymes [4]. Iron exists in nearly all living entities, particularly archaea, bacteria, plants, and humans and it plays a crucial role in various biological activities. Iron is an essential micronutrient in fish and is involved in critical activities such as Fe-S cluster formation. It also has antimicrobial properties against numerous fish pathogens [5]. Iron-based materials might be useful in the real world. Iron oxide, which constitutes a significant proportion of iron-based nanomaterials, has excellent superparamagnetic properties and catalytic activity mimicking oxidoreductases [6]. Magnetic iron oxide nanoparticles (IONPs), particularly superparamagnetic magnetite (Fe₃O₄), represent the most prospective and extensively utilized class of magnetic nanoparticles (MNPs) in comparison to α-Fe₂O₃ (hematite) and γ-Fe₂O₃ (maghemite) for biomedical applications. Their primary advantages encompass enhanced biocompatibility, minimal cytotoxicity, and exceptional magnetic characteristics. These properties render IONPs, especially Fe₃O₄, highly appropriate for sophisticated medical applications, thereby stimulating continued research aimed at their commercialization for wider clinical adoption [7, 8]. Additionally, iron-based sulfides have developed as a category of iron-based materials and received significant interest as possible candidates for development and application in the biomedical sector. It’s important to know that the iron atoms in iron-sulfide molecules could interact with the sulfhydryl groups on cysteine in living things to create a ferromagnetic and antiferromagnetic iron sulfide cluster. On the other hand, iron-based sulfides can overcome the conflict between dependable biosafety and exceptional multifunctionality, opening up novel ways for effective biomedical uses [9]. Iron sulfide nanomaterials, a type of congeneric element, are still not extensively investigated or utilized in biomedical sectors. They exhibit almost identical physiochemical characteristics to iron oxide [10], with phases like pyrite (FeS_2_), mackinawite (FeS), greigite (Fe_3_S_4_), and pyrrhotite (Fe_1−x_S). Iron sulfide’s smaller band gap allows for better electron transfer and conductivity [11]. Iron-sulfur aggregates are crucial cofactors in numerous enzymes, serving as active sites for electron transmission in respiratory chain reactions and catalytic processes [12]. However, the urgent need for a quicker, cheaper, more productive, non-toxic, and environmentally friendly procedure has turned attention to greener methods. Researchers are exploring green production of metal nanoparticles utilizing plants like *Ephedra* [13] and *Olea europaea* [14], which are sustainable and effective. Unlike previous methods, this eco-friendly technology incorporates bioactive compounds as natural reducing and stabilizing agents. Researchers have synthesized iron nanoparticles from plant leaves, fruit, and pits with extensive biological activities for biomedical applications. For instance, Nigam and Kala [15] synthesized iron sulfide nanoparticles using green means in two steps: Fe nanoparticles were produced in solution by reducing iron salt with *Urtica dioica* leaf extract, and then Na₂S was added at room temperature to convert them into nanoparticles with antibacterial and antifungal properties. Likewise, microorganisms have been found to be effective in the biosynthesis of iron sulfide for biomedical purposes [16]. They formed iron sulfide nanoparticles on the surface, allowing normal metabolism without preventing the porous structure, proving the method’s efficiency in NP production [17]. This approach enhances the biocompatibility of iron sulfide, resulting in increased catalytic reactivity and a larger surface area. Although making nanoparticles from biological sources, via bacteria, actinobacteria, algae, fungi, and yeast, is considerably safer than using chemical and physical processes [18]. Bacteria are favored over other microorganisms for the production of nanoparticles because they may be cultivated successfully in artificial environments with an optimum growth rate. Many fungi have been found to create nanoparticles of various shapes and sizes. However, there are several obstacles to produce nanoparticles from fungi, such as lengthy production processes and expensive downstream processing, which must be focused on to develop practical methods for producing nanoparticles on a large scale at a reasonable cost [19]. FeS nanoparticles are representative iron-based sulfides that can be predominantly biosynthesized through sulfate-reducing bacteria (SRB) in anoxic environments. In previous studies, evidence can be found that the production of FeSNPs is related to the metabolic process of SRB, which reduces sulfate (SO₄²⁻) as an electron acceptor to form S²⁻ [20]. The regulated manufacturing of biogenic FeSNPs can be performed by systematically adjusting these mechanisms [21]. Prior research indicates that the conversion rate of precursors is essential to the dimensions of biogenic nanoparticles. In this case, the overall precursor conversion can be regulated by adjusting the initial dosages of iron and sulfur precursors, resulting in FeSNPs with distinctive size and crystal structure [22]. Uncultured magnetotactic bacteria produce Fe₃S₄. In these uncultured cells, magnetosomes were long and rectangular. The cell had about 40 magnetosomes, mostly in a big cluster. Chain-like magnetosomes were also seen near the main cluster [17]. However, which biological groups, signaling pathways, and protein expressions are involved during the reaction remains unclear. Therefore, in-depth exploration of biological mechanisms is urgently needed and will contribute to the green controllable synthesis of related materials on morphological and structural scales [23]. Gram-positive aerobic endospore-forming *Bacillus sonorensis* is a *Bacillus subtilis* member. It was isolated from Sonoran Desert soil and is phenotypically and genotypically similar to *Bacillus licheniformis*, showing facultative anaerobic characteristics [24]. *B. sonorensis* and *B. licheniformis* can be discriminated by colony pigmentation [25] and clindamycin sensitivity [26]. *B. licheniformis* sp. is used for the commercial manufacture of enzymes, antibiotics, biochemicals and as a host for cloning α-amylase genes. Since the industrially relevant bacterium *B. licheniformis* is closely related to *B. sonorensis*, which is less known, the genome of *B. sonorensis* L12, which was isolated from Gergoush’s main starting materials in Sudan, was sequenced [25]. The information gained was used to investigate the biotechnological importance of this organism, its genomics, and its phylogenetic status relative to *B. subtilis*, which will improve our understanding of *B. sonorensis.* Initial genome research showed that the strain *Bacillus sonorensis* L12 draft genome sequence is 4,647,754 bp and 45.2% G + C. This strain has gene clusters for de novo biosynthesis of the nonribosomal lipopeptides fengycin, iturin, and bacitracin, which may have biotechnological applications [27]. It is a viable strain for cloning to develop and produce novel antibacterial compounds and as a bioprotective agent to combat plant fungal infections in agricultural farming. Comparative genomic investigations will also illuminate this species’ evolutionary and phylogenetic status and improve our understanding of its ecology and evolution [28]. In 2015, Nerurkar et al. [29] produced lipase from the newly isolated marine bacterium *Bacillus sonorensis* from the clams *Paphia malabarica* found in the Kalbadevi estuary, Mumbai, India, and compared it to the alkaline scouring of cotton fabric. The study confirmed that *B. sonorensis* lipase could hydrolyze wax into fatty acids and scour cotton fibers. Bioscouring with lipase at 8% on fabric weight (owf) at pH 9, 60 °C, and 120 min exhibited the highest weight loss and hydrophilicity. Bioscouring with lipase from *B. sonorensis* was as successful as alkaline treatment in wettability, whiteness, dyeing behavior, tensile strength, and bending stiffness. Furthermore, Hyperthermostable alkaline lipase from *Bacillus sonorensis* 4R was isolated and described by Bhosale et al. [30]. This enzyme synthesis occurred in glucose-tween inorganic salt broth at 80 °C and pH 9 for 96 h under static circumstances. The enzyme activity peaked at 80 °C with a t_1/2_ of 150 min, followed by 90 °C, 100 °C, 110 °C, and 120 °C with values of 121.59, 90.01, 70.01, and 50 min, respectively. The enzyme was substantially activated by Mg, and adding magnesium and mannitol together enhanced t_1/2_ values at 80 °C from 150 to 180 min. Lipase activity peaked at pH 9 and lasted 2 h at 80 °C. In addition, *Bacillus sonorensis* T6, a soil-derived xylanolytic bacterium, was isolated by Kiribayeva et al. [31]. The *Bacillus sonorensis* T6 xylanase gene was cloned and expressed in *Escherichia coli* and *Pichia pastoris* to produce rXynT6-E and rXynT6-P enzymes. Recombinant xylanases performed best at 47–55 °C and pH 6 − 7. The *P. pastoris* recombinant xylanase is 40% more thermally stable than the *E. coli* one. Recombinant xylanases retained 100% activity after 10 h incubation at pH 3–11 and 68% after 1 h at pH 2. Superior stability in a wide pH range and moderate temperatures may make *Bacillus sonorensis* T6 xylanase ideal for biotechnological applications. The available sources provide limited information on *B. sonorensis* unique capabilities for magnetic nanoparticles synthesis, focusing instead on other *Bacillus* species. The previous literature does not provide any precise information about *Bacillus sonorensis* magnetic nanoparticle manufacturing capabilities. However, other *Bacillus* species, such as *Bacillus subtilis* SE05, *B. velezensis* SMR, and *Bacillus* sp., have been intensively explored for the biosynthesis of iron oxide nanoparticles. A study by Abouelkheir et al. [32] describes a straightforward, biologically and environmentally friendly method for creating magnetic iron oxide (γ-Fe₂O₃) nanoparticles. The *Bacillus subtilis* SE05 strain, isolated from Egypt, can produce highly magnetic iron oxide nanoparticles of the maghemite type (γ-Fe₂O₃). The ability of this bacterium to reduce Fe₂O₃ has never been demonstrated before. An experimental study by Elsharkawy et al. [33] examined the dual modulation of the colchicine-induced rat model of Alzheimer’s disease by superparamagnetic iron oxide nanoparticles (SPIONs) produced by *B. velezensis* SMR and the soluble product of the *Dipylidium caninum* adult worm. Another study by Abouelkheir et al. [34] used the red tilapia (*Oreochromis* sp.) as a model organism to talk about the subtle cellular alterations caused by the biologically induced biomineralized Fe₃O₄-SPIONs by *Bacillus* sp. in the early-life stages. These species often reduce metal ions into nanoparticles, either intracellularly or extracellularly, with enzymes and other secreted biomolecules serving as reducing and capping agents [35]. Various methodologies and procedures are employed to analyze and characterize the physical and chemical characters of these nanoparticles, resolving specific properties and features and determining their potential applications [36–39]. Cancer treatment has evolved rapidly, with new limited negative effects and exceptionally high specificity treatments. Inorganic nano-platforms can intelligently respond to specific microenvironments and activate the immune system, leading to more accurate and effective treatment. Iron-based sulfides, with chemodynamic properties and enhanced absorbance in the near-infrared spectrum, exhibit prospective applications in tumor treatment, involving chemotherapeutic and photothermal treatment. Combining these nano-platforms with physical tools can activate the immune system and improve treatment outcomes [9]. The ability of FeSNPs to affect many carcinogenic pathways has made them promising therapeutic agents for the treatment of cancer. FeSNPs induce apoptosis, inhibit angiogenesis, and disrupt critical signaling pathways that facilitate tumor growth, all of which enhance their potent anticancer properties. Studies have demonstrated that FeSNPs induce cancer cell death by facilitating Fe²⁺ release, increasing reactive oxygen species (ROS) levels, consuming glutathione (GSH), and promoting lipid hydroperoxide (LPO) accumulation. These biochemical interactions contribute to oxidative stress and ferroptosis, ultimately impairing cancer cell survival. As a result, FeSNPs exhibit outstanding therapeutic efficacy in vivo, highlighting their potential as a viable approach for cancer therapy [40–42]. Future studies are required to further explore FeSNPs pharmacokinetics, bioavailability, and clinical applicability in cancer therapy. To our knowledge, the physicochemical properties of FeSNPs, such as size, shape, and surface coating or functionalization, significantly influence their pharmacokinetics and bioavailability. To fill the gap in our knowledge about the physicochemical properties and bioavailability of FeSNPs, this study aims to biologically synthesize superparamagnetic iron sulfide nanoparticles (FeSNPs) and explain their physicochemical properties. This investigation provides important evidence needed for their potential use as new multifunctional biomaterials in medicine, particularly in cancer therapy.

## Materials and methods

### Materials

All media components were obtained from Oxoid. Fetal bovine serum and Dulbecco’s Modified Eagle’s Medium (DMEM) containing high glucose and pyruvate were purchased from Gibco Laboratories, Mumbai, India. Dimethyl sulphoxide (DMSO) was purchased from HiMedia (Mumbai, India). 3-(4,5-dimethylthiazol-2-yl)-2,5- diphenyltetrazolium bromide (MTT) was purchased from Sigma-Aldrich Corporation, Bangalore, India. Human breast cancer (MCF-7), human lung adenocarcinoma of alveolar basal epithelial (A549), human hepatoma (HepG2), human immortal cell line (Hela), and colorectal carcinoma (HCT-15) were obtained from the Naval Medical Research Unit 3, Cairo, Egypt. All additional substances used were of high analytical grade.

### Isolation and screening procedures

Marine sediment samples were collected in sterile screw bottles. Samples were prepared using the serial dilution technique. Bacteriological medium nutrient agar was utilized for isolation, screening, and seed culture preparation, which contained g/L peptone (5), beef extract (3), NaCl (5), and agar (20). The pH was adjusted to 7 with NaOH. One milliliter from each dilution (1 mL) was aseptically dispensed in agar plates and incubated at 60 °C. Apparently, ten different colonies were picked up and purified according to the standard microbiological purification methods [32]. The ten purified isolates were screened for their biosynthesis ability of iron sulfide nanoparticles by being transferred to 100 mL nutrient broth (NB) culture media with the same components as nutrient agar medium without agar and incubated at 120 rpm for 24 h. The whole culture was supplemented with an equal volume of salt solution (1 mM) of ferrous sulphate heptahydrate (FeSO₄0.7 H₂O) (Nice Chemicals, Egypt) as a substrate. The reaction mixture was incubated for another 24 h on a rotary shaker at 60 °C and 120 rpm. The production of iron sulfide nanoparticles was observed by the solution’s color transformation to black.

### Marine bacterial identification

The genomic DNA of the promising bacterium was extracted in accordance with the manufacturer’s protocols utilizing the DNeasy Blood and Tissue Kit (Qiagen, Hilden, Germany). Using a set of universal primers, 27 F (5′- AGAGTTTGATCCTGGCTCAG − 3′) and 1494 R (5′- TACGGCTACCTTGTTACGAC − 3′), the 16S rRNA genes were amplified [43]. Thermal cycling was performed as follows: template DNA 1 µL, forward primer (10 µM) 1 µL, reverse primer (10 µM) 1 µL, KODone master mix 25 µL, and H_2_O to a final volume of 50 µL. The PCR condition was 95 ˚C 1 min – (95 ˚C 15 s, 50 ˚C 15 s, 68 ˚C 10 s) x 30–68 ˚C 1 min. 1% (w/v) agarose gel electrophoresis was employed to verify the amplification products. PCR reaction solution was purified by kit (Wizard^®^ SV Gel and PCR Clean-Up System, Promega). DNA sequence analysis was performed by Fasmac Company (https://fasmac.co.jp/en). The sequences were processed and edited by BioEdit software (Version 7.2) and aligned with those in the National Centre for Biotechnology Information (NCBI) database through the BLASTn program1.

### Biotic synthesis of iron sulfide nanoparticles (FeSNPs)

For the biosynthesis of nano-sized iron sulfide (FeSNPs), the *B. sonorensis* SS1 strain was cultured in 100 mL of nutrient broth medium and incubated at 60 °C for 24 h in a shaking incubator at 120 rpm. An equal volume of the whole culture was added to a 50 mM ferrous sulphate (FeSO_4_.7H_2_O) substrate and incubated again in an orbital shaker at 120 rpm and 60 °C for another 24 h. The nanoparticle synthesis was validated through visual monitoring of the colour shift to black. The biosynthesized FeSNPs were collected using an external magnet. The purified nanoparticles were washed using distilled water and subsequently allowed to dry in a hot air incubator for characterization and the rest of the investigations [32, 44, 34].

### Nano-sized iron sulfide (FeSNPs) characterization

The biogenic nano-sized iron sulfide (FeSNPs) were characterized regarding their size, morphology, and magnetic properties using various techniques including XRD, TEM, EDX, FTIR, zeta potential, VSM, and BET. The XRD diffractogram and the crystal phase of FeSNPs nanoparticles were acquired using an X-ray powder diffraction-XRD-D2 Phaser (Bruker, Germany) with a copper X-ray source operating at 30 kV and 10 mA. Scans were recorded at a rate of 2° per min within the 2θ range of 10° to 100°. The crystallite size was assessed based on Scherrer’s Eq. (1) [34].

1 $$\boldsymbol{C}\boldsymbol{r}\boldsymbol{S}=\mathbf{K}\boldsymbol{\uplambda}/\left(\boldsymbol{\upbeta}\mathbf{cos}\boldsymbol{\uptheta}\right)$$

where (k) represents the dimensionless Scherrer constant = 0.94, (λ) corresponds to the X-ray wavelength = 1.54184 nm, (β) signifies the peak full width at half maximum in radians, and (θ) indicates the diffraction angle in radians. The EDX spectra of the purified iron sulfide nanoparticles were measured with a scanning electron microscope (SEM) (Jeol JSM-IT 200). They were characterized based on size and morphology using transmission electron microscope (TEM). An FTIR examination was conducted via a Bruker Tensor 37 FTIR Spectroscope. The spectral range extended from 4,000 to 500 cm^− 1^, with the signal acquired at a resolution of 1 cm^− 1^. Nanoparticles were characterized in terms of zeta potential via the Malvern Zetasizer Nano ZS, Malvern Instruments, (Malvern, UK) following dilution with deionized water. The magnetic characteristics of iron sulfide (FeSNPs) nanoparticles were determined through a Vibrating Sample Magnetometer (VSM). The surface features of the synthesized FeSNPs were derived from N2 adsorption-desorption isotherms obtained at -196 °C with BELSORP max II equipment, Japan. The particles were first outgassed under vacuum (10 − 4 Torr) at -195.790 °C. BET surface areas (SBET) were determined using the BET equation. Pore size distributions (PSD) were determined by applying the Barrett, Joyner and Halenda (BJH) technique.

### Medical applications of nano-sized iron sulfide (FeSNPs)

#### Antagonistic effect on fish pathogens

The study used the agar-well diffusion procedure to analyze the antagonistic activity of FeSNPs. Muller Hinton Agar plates were inoculated with three pathogen cultures: *Pseudomonas fluorescens* Migula 1895^AL^ (ATCC 13525), *Aeromonas hydrophila* (ATCC 13037), and *Streptococcus agalactiae* Lehmann and Neumann 1896^AL^ (ATCC 13813), provided from the Microbiological Resources Center (Cairo MIRCEN), Faculty of Agriculture, Ain Shams University, Egypt. 50 µL of a nano-sized iron sulfide (FeSNPs) - dimethylsulfoxide (DMSO) solution (500 µL FeSNPs:500 µL DMSO) was added to MHA plates, with DMSO as a negative control, and the diameter of the clear zone of inhibition was documented after 24 h of cultivation at 37 °C. The minimum inhibitory concentration (MIC) and minimum bactericidal concentration (MBC) of FeSNPs against *Aeromonas hydrophila* (ATCC 13037) were determined using the broth microdilution method. A bacterial suspension adjusted to 0.5 McFarland standard was diluted in Luria–Bertani (LB) broth to obtain a final inoculum of approximately 5 × 10⁸ CFU/mL. FeSNPs were dispersed in sterile deionized water by ultrasonication for 30 min, followed by two-fold serial dilutions in LB broth to achieve final concentrations of 12.5, 25, 50, 75, and 100 µg/mL in sterile 96-well microtiter plates. Each well was inoculated with 50 µL of the bacterial suspension and incubated at 37 °C for 24 h [45]. The MIC was defined as the lowest FeSNPs concentration showing no visible bacterial growth. For MBC determination, 10 µL aliquots from wells without visible growth were plated onto LB agar and incubated at 37 °C for 24 h, and the MBC was recorded as the lowest concentration resulting in complete absence of colony formation. All assays were performed in triplicate, and reproducible results were obtained.

#### Cytotoxicity against cancer and normal cell lines (in vitro)

The cytotoxicity of green synthesized FeSNPs was assessed through the reduction of tetrazolium by the MTT (3-(4,5-dimethylthiazol-2-yl)-2,5-diphenyl tetrazolium bromide) assay [46]. Five tumor cell lines namely Human cancer cell lines MCF-7 (breast carcinoma), A549 (lung carcinoma), HeLa (cervical carcinoma), HepG2 (hepatocellular carcinoma), HCT-116 (colorectal carcinoma) and one normal cell line PBMCs (Peripheral blood mononuclear cells) were used. Briefly, cells were inoculated at a density of 5 × 10³ cells per well (25 µL cell suspension) in 96 wells containing Dulbecco’s Modified Eagle’s Medium with high glucose and pyruvate, and cultivated at 37 °C for 48 h in 5% CO_2_ to establish a monolayer. This layer of cells was supplemented with different doses of FeSNPs (12.5, 25, 50, 75 and 100 µg/mL) prepared in DMSO. Following overnight cultivation at 37 °C, cells were centrifuged, the supernatant was discarded and 200 µL of MTT was incorporated into each well and maintained at 37 °C for 4 h. The resulting formazan crystals were dissolved in 100 µL of DMSO. Tumor and normal cells incubated alone with DMSO served as the control. Metabolically active cells with undamaged mitochondria transform yellow tetrazolium MTT into a water-insoluble purple formazan crystal. The quantification of MTT reduction was performed spectrophotometrically at 545 nm. The relative cell viability was determined by the absorbance ratio of the test sample to that of the nanoparticle concentration that showed toxicity to various cells [47]. All experiments were carried out in triplicate (*n* = 3 independent biological replicates) and all data are presented as mean ± standard deviation (SD). Statistical significance was assessed using one-way ANOVA followed by the Duncan post hoc test, with *p* < 0.05 considered statistically significant. IC₅₀ values were determined from dose-response curves using GraphPad Prism software (Version 9.0, GraphPad Software, San Diego, CA, USA).

### Molecular modeling analysis

Protein Data Bank 3D structures of Caspase-3 (PDB ID: 2XYG) [48], VEGFR (PDB ID: 4AGD) [49] and Aurora A (PDB ID: 4DEB) [50] were acquired. The “Prepare Protein” function in DS enabled the addition of any missed atoms or chains while eliminating water molecules, making the protein structure ready for docking simulations. A binding sphere encompassing the co-crystallized ligand was selected to determine the binding site. Subsequently, employing the default configurations of CDOCKER [51], a grid-based docking tool, the active compounds were docked into the binding sites of Caspase-3, VEGFR and Aurora A. To account for the size disparity between the nanoparticle and the protein active sites, docking was performed using an enlarged grid (30 × 30 × 30 Å). This allowed for the simulation of multi-point surface binding rather than rigid occupancy. Eventually, the most advantageous site of the docked compounds was specified. Three-dimensional (3D) figure presentations were accomplished with PyMOL software [52].

#### In vitro enzyme assays

Caspase-3 (Active) ELISA assay quantification of active Caspase-3 was performed using the Invitrogen Caspase-3 (active) Human ELISA Kit [53]. Cell Lysis: Cells were lysed using a protease-inhibitor-supplemented buffer for 30 min on ice. Incubation: 100 µL of standards and samples were added to antibody-coated wells and incubated for 2 h at room temperature. Detection: Wells were incubated with a rabbit anti-active Caspase-3 detection antibody (1 h), followed by Anti-Rabbit IgG HRP (30 min). Quantification: TMB substrate was added, and the reaction was stopped after 30 min using a stop solution. Absorbance was read at 450 nm. VEGFR2 Kinase assay: The VEGFR2 (KDR) Kinase assay kit was used to measure inhibitory activity via luminescence [54]. Reaction Mixture: Purified VEGFR2 enzyme (1 ng/µL) was incubated with ATP, PTK substrate, and the test compound in 1x Kinase Buffer at 30 °C for 45 min. Detection: Kinase-Glo MAX reagent was added to each well and incubated for 15 min to measure remaining ATP levels. Reference: Sorafenib served as the positive control. Aurora A Kinase inhibition assay activity was verified using a recombinant human Aurora A GST-fusion protein. Reaction: 50 ng of Aurora A was incubated with biotinylated peptide substrate and 200 µM ATP for 30 min at room temperature [55]. Detection: Phosphorylation of the substrate was detected using a phospho-Serine antibody and measured via DELFIA^®^ time-resolved fluorescence (Ex: 340 nm, Em: 615 nm). Reference: Danusertib was used for comparative inhibition analysis.

### Data statistical analysis

The study involved three replicates of each experiment, and statistical data analysis was done using ImageJ and Origin Pro 8.1, with P-values of 0.05 indicating significant results.

## Results

### Biosynthesis of FeSNPs by *Bacillus sonorensis* SS1

**Fig. 1:**
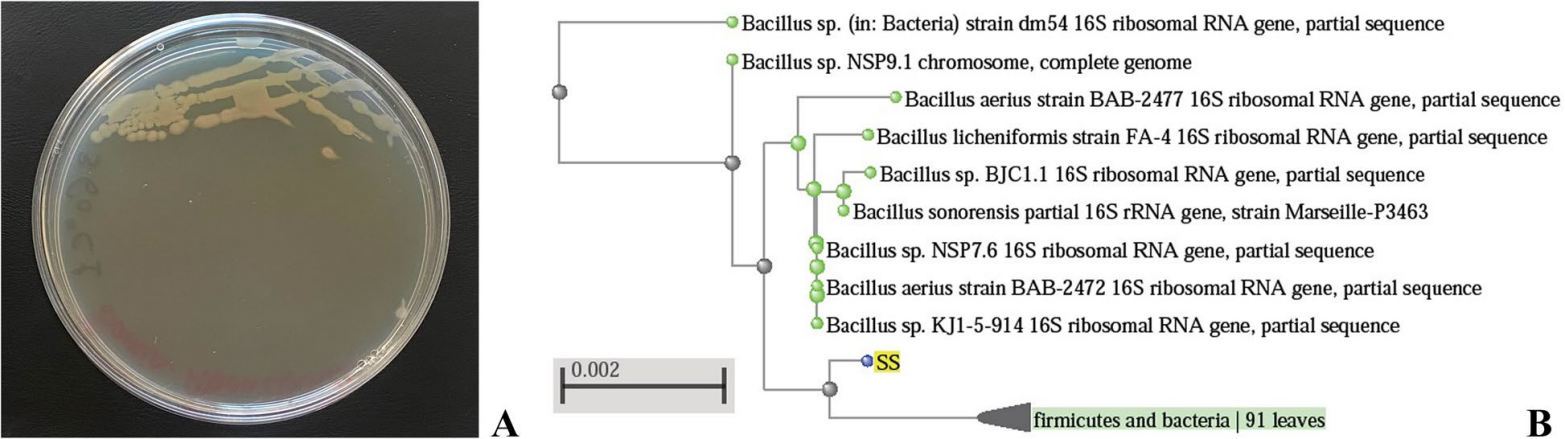
Iron sulfide nanoparticles producing *Bacillus sonorensis* SS1: colonies on nutrient agar (**A**); Neighbor joining phylogenetic tree derived from 16S rRNA gene sequencing illustrating the evolutionary linkage between *Bacillus sonorensis* SS1 and similar* Bacillus* species (**B**)

*Bacillus sonorensis* SS1 (Fig. 1A), an endospore-forming, thermophilic, and nanoparticle-producing strain, was isolated from marine sediment in Egypt. The molecular identification was recognized through total DNA extraction and PCR on the 16S rRNA gene. The 16S rRNA gene was successfully amplified, resulting in a length of 1449 bp. The sequence was compared to other strains and submitted to the GenBank of the NCBI under the accession number SS1 OQ195211. The phylogenetic tree was generated via the neighbor-joining algorithm; the phylogram analysis clarified the genetic divergence among the sequences and confirmed the sequence’s identity as *Bacillus sonorensis* with 100% similarity (Fig. 1B).

**Fig. 2:**
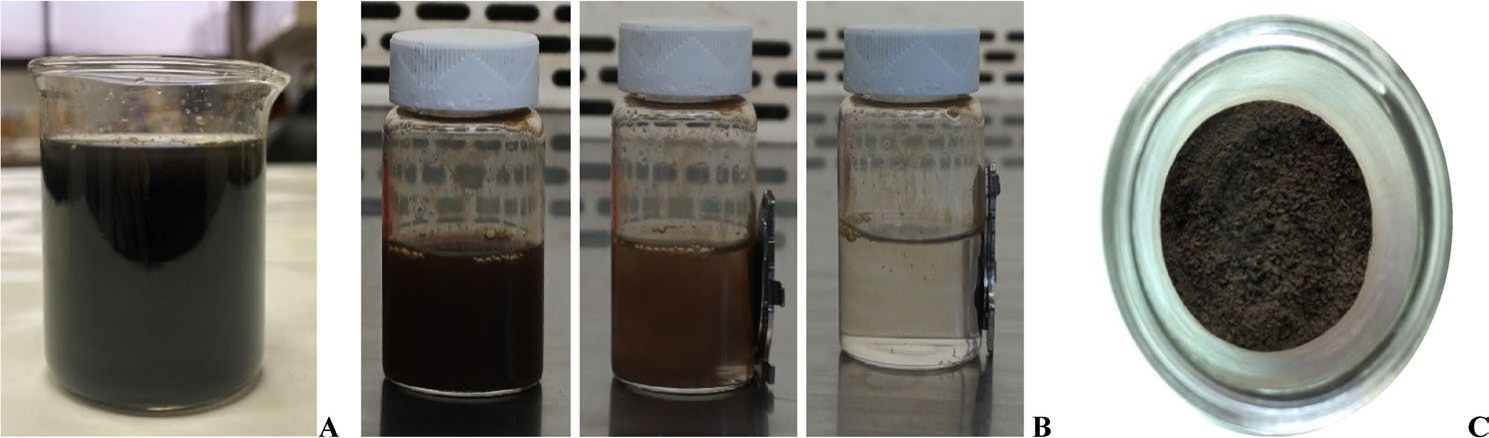
Biosynthesis, extraction, and purification of iron sulfide nanoparticles: FeSNPs in colloidal solution (**A)**; purification using external magnet (**B)**; purified black nanopowder (**C)**

Thermophilic bacteria can be considered promising agents for synthesizing highly improved industrially relevant nanomaterials. The purified black nanopowder (FeSNPs) produced (10.62 g/L) via a simple biological method by *Bacillus sonorensis* SS1 (Fig. 2A, B, C) was stable at elevated temperatures (60 °C) and pH values (pH 9), suggesting its potential use in high-temperature and alkaline processes in the industry. This study investigated synthesis reproducibility by managing crucial reaction parameters, such as pH, temperature, stirring rate, and the use of the same batch of biological reducing agents, to reduce or eliminate fluctuations in reducing power.

### Characterization of FeSNPs nanoparticles

**Fig. 3:**
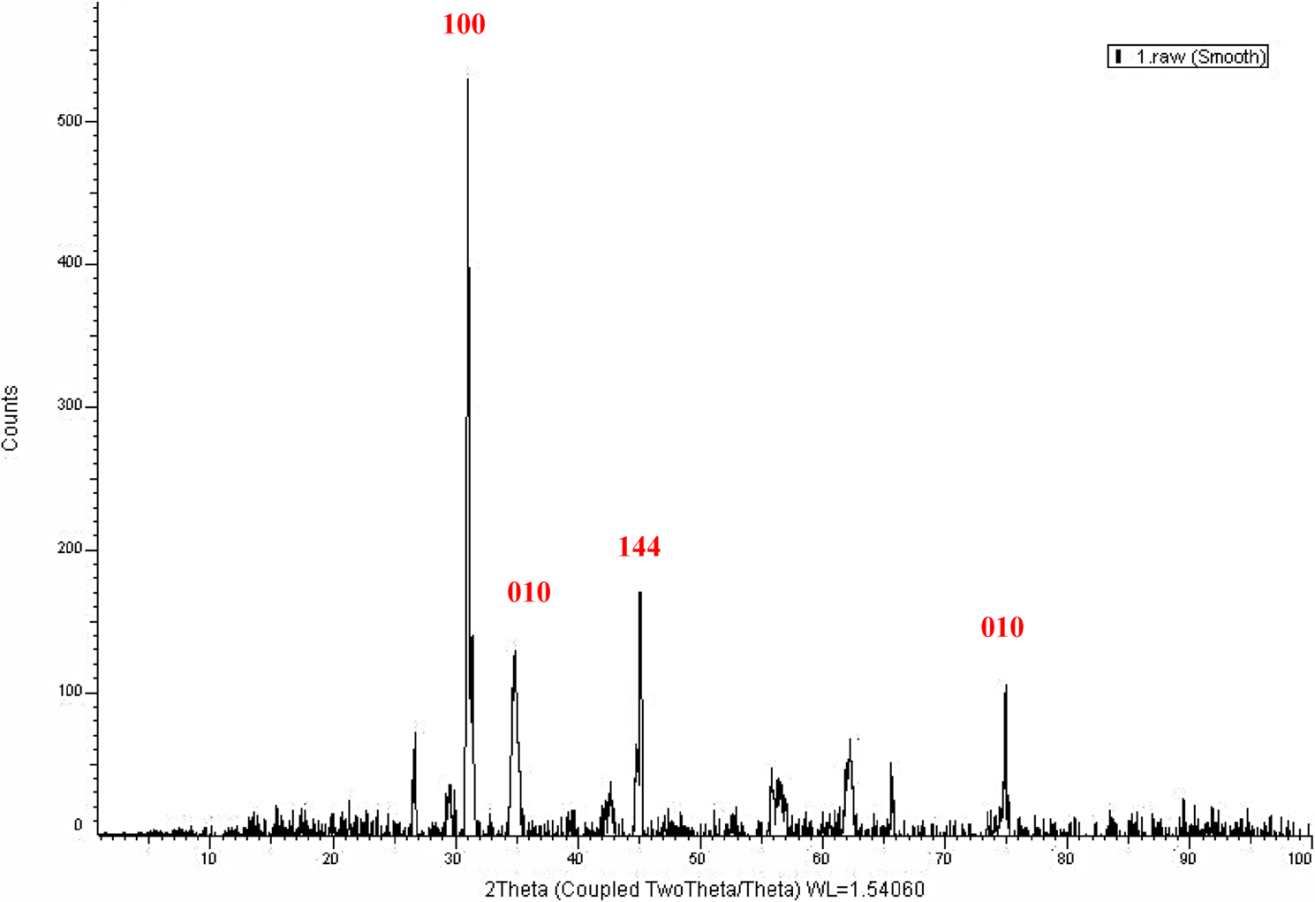
X-ray diffraction patterns (XRD) of iron sulfide nanoparticles (FeSNPs)

The powder X-ray diffraction (XRD) patterns of the iron sulfide nanoparticles (FeSNPs) are displayed in Fig. 3. The X-ray diffraction patterns revealed ten peaks with four major reflections at peak positions at 2θ equal to 31.0°, 35.1°, 45.2°, and 75.0°, corresponding to (100), (010), (144), and (010). The four main diffraction peaks could be correlated with the standard diffraction data of the crystalline phase of iron sulfide with the chemical formula Fe_0.985_S (the Inorganic Crystal Structure Database (ICSD) Collection Code: 068849 [56] and Fe_1-x_ S [57]. Using Scherrer Eq. (**1**), we found that the crystallite sizes of the four main reflections were 17 nm, 17 nm, 18 nm, and 20 nm, with a notable peak at 31.0° at 17 nm. The strong crystalline structure (Hexagonal lattice structure) of the produced FeS nanoparticles is shown by the tall peaks that appeared in the analysis (Fig. 3).

**Fig. 4:**
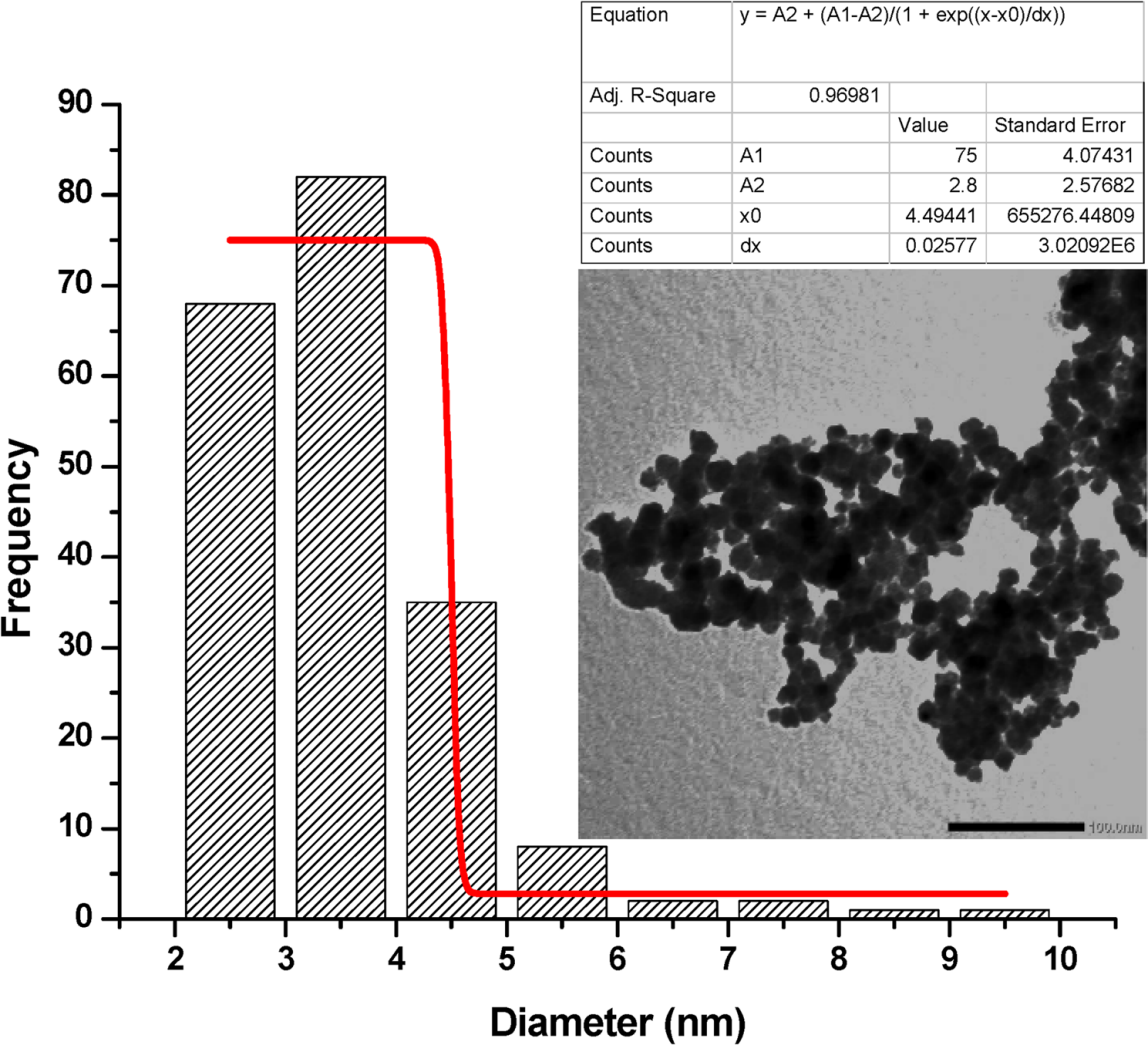
Transmission electron microscope (TEM) exploration of iron sulfide nanoparticles (FeSNPs) shows the distribution of nanoparticles at a higher magnification of 100 nm showing the range of particle sizes with average of 4.5 nm

Transmission electron microscope (TEM) revealed, as illustrated in Fig. 4, extremely dispersible spherical nanoparticles from the properly prepared FeSNPs. Size distribution curve from TEM scans revealed that FeSNPs measured an average size of 4.5 nm. EDX analysis demonstrated the purity of the sample and revealed the identification and quantification of the elements found in the produced FeSNPs.

**Fig. 5:**
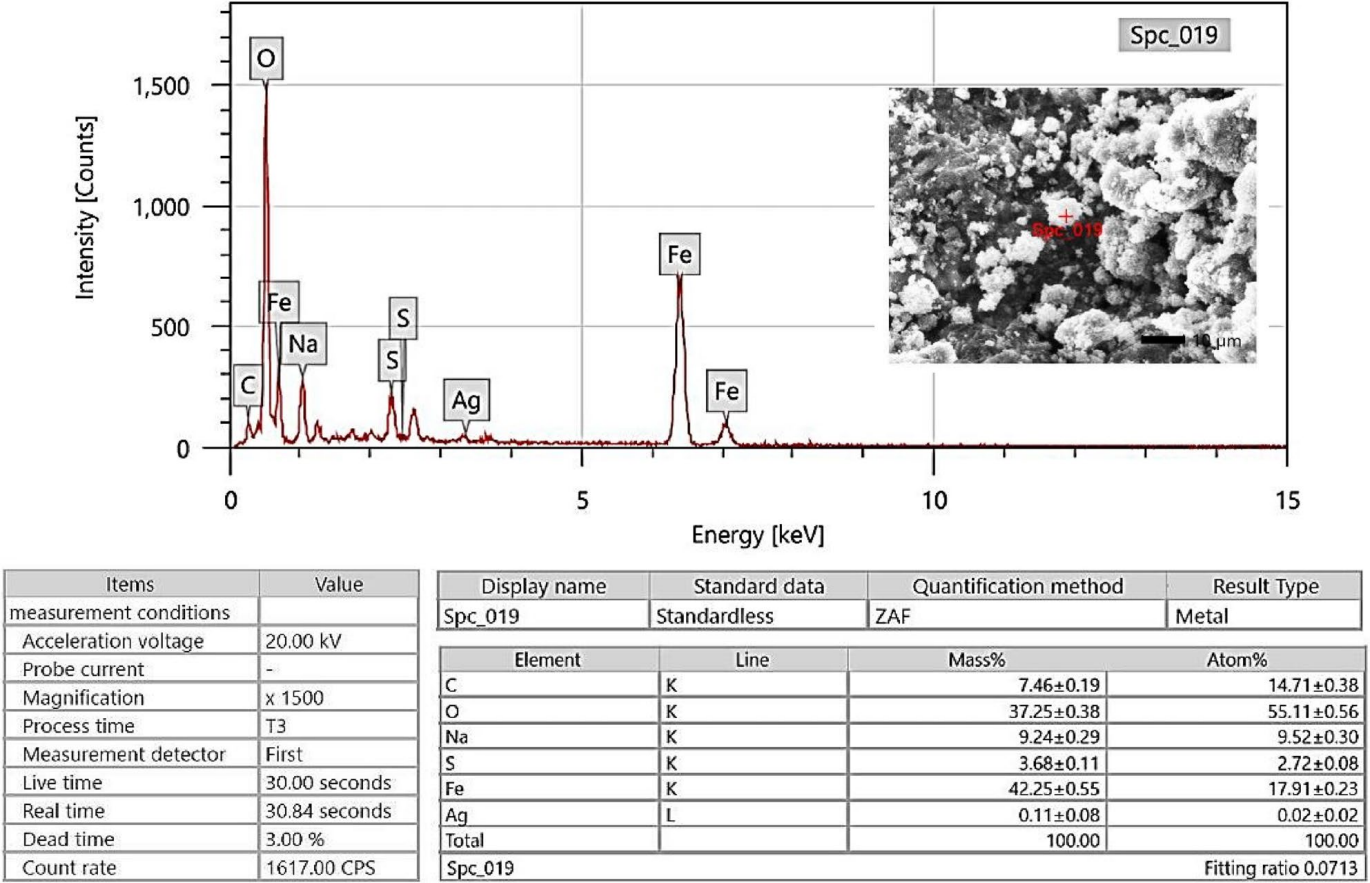
Elemental analysis using energy dispersive x-ray (EDX) spectrometry

The EDX analysis results depict that the weight proportion of iron particles constitutes 42.25 wt% of the total weight, whereas sulfur particles accounts for 3.68 wt%. Oxygen accounts for 37.25 wt% of the total weight, sodium accounts for 9.24 wt%, carbon accounts for 7.46 wt%, and silver accounts for 0.11 wt% (Fig. 5).

**Fig. 6:**
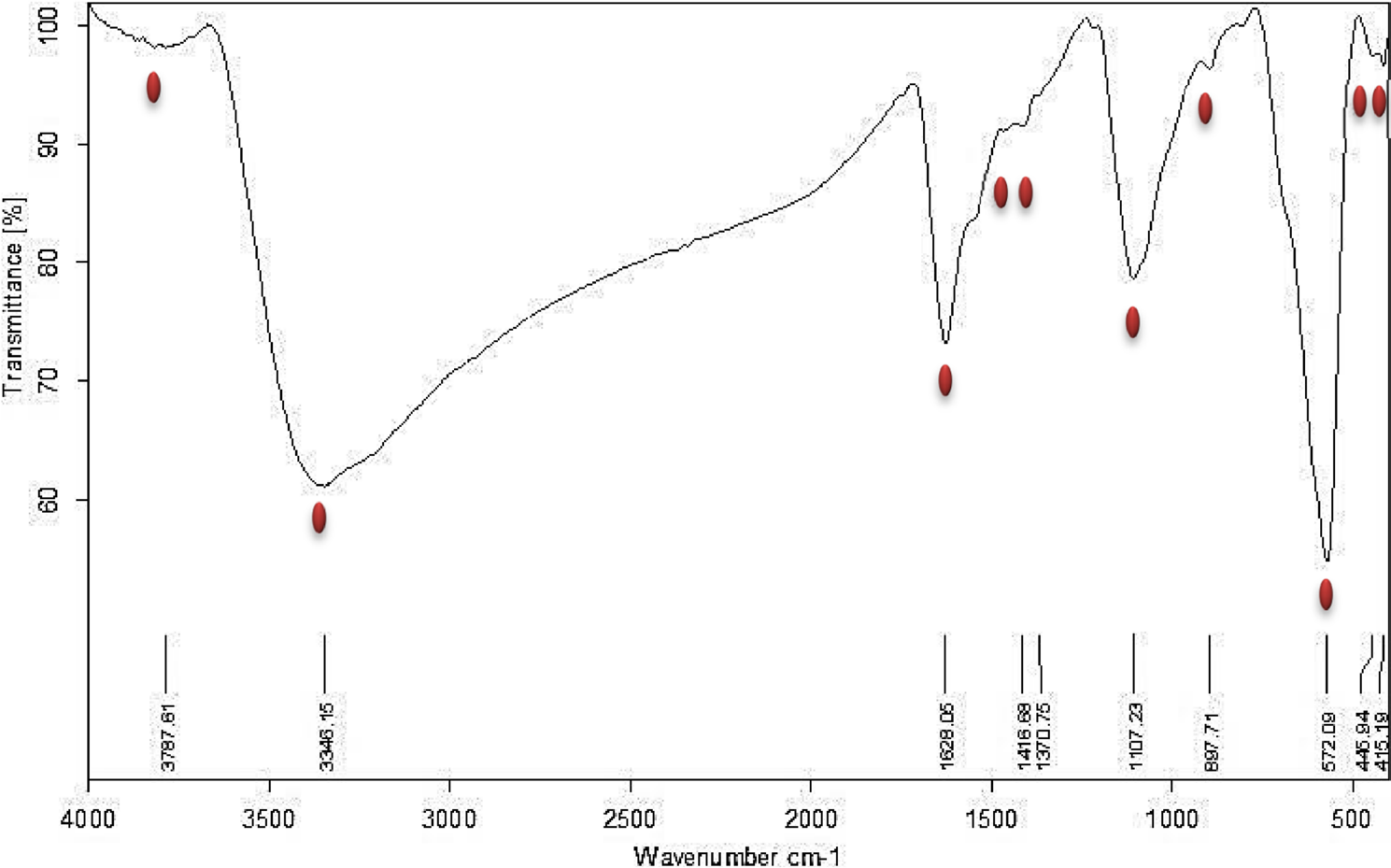
FTIR analysis of biosynthesized FeSNPs

During the biological synthesis of the FeSNPs nanoparticles, they are capped by several functional groups as demonstrated by FTIR analysis (Fig. 6). These functional groups (Table 1) act as surface ligands to improve their biocompatibility. The FeSNPs’ IR spectrum shows some unique peaks at 3787.6131, 3346.1489, 1628.0517, 1416.6823, 1370.7494, 1107.2268, 897.7119, 572.0903, 445.9431, and 415.1934 cm⁻^1^. These peaks correspond to O–H (alcohol), N–H (primary and secondary amines, amides), C = C (alkene), S = O (sulfite) / O-H (alcohol), N-O (nitro compound) / C–H (alkane), C–O (secondary alcohol), C–H (1,2,4- trisubstituted), C-Br (halo compound), C-I (Alkyl and Aryl Halides), and C-I (Alkyl and Aryl Halides), respectively.

**Table 1:** FTIR spectra and the peak positions of the major IR bands of FeSNPs

Peak wavenumber of FeSNPs cm^− 1^	Bond/Compound Class	Standard peak reference
3787.6131	O–H stretching, alcohol	[58]
3346.1489	N–H stretch/primary, secondary amines, amides	[58]
1628.0517	C = C stretching, alkene	[58]
1416.6823	S = O, Sulfite / O-H, bending, alcohol	[58]
1370.7494	N-O, stretching, nitro compound / C-H, bending, alkane	[58]
1107.2268	C-O, stretching, secondary alcohol	[58]
897.7119	C–H, bending, 1,2,4- trisubstituted	[58]
572.0903	C-Br, stretching, halo compound	[59]
445.9431	C-I, stretching, Alkyl and Aryl Halides	[60]
415.1934	C-I, stretching, Alkyl and Aryl Halides	[60]

**Fig. 7:**
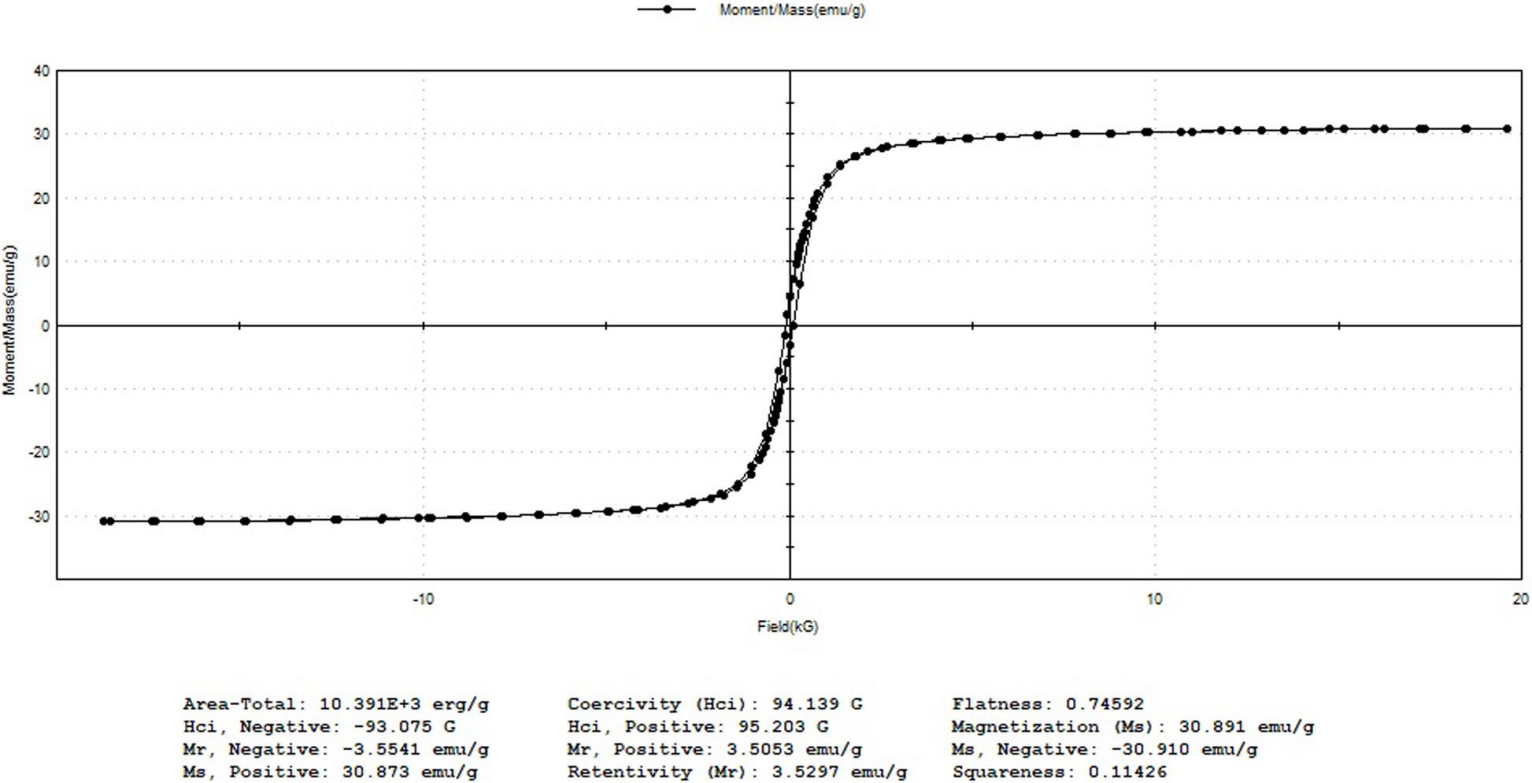
Vibrating sample magnetometer (VSM) chart of biosynthesized FeSNPs

The magnetic properties of the biosynthesized FeSNPs nanoparticles were characterized by VSM at 300 K (Fig. 7). Well-defined ferromagnetic hysteresis loops were obtained up to ± 20 kOe. The measured saturation magnetization (Ms) was 30.873 emu/g. The coercivity (Hci) was 94.139 G and the retentivity (Mr) was 3.5297 emu/g.

**Fig. 8:**
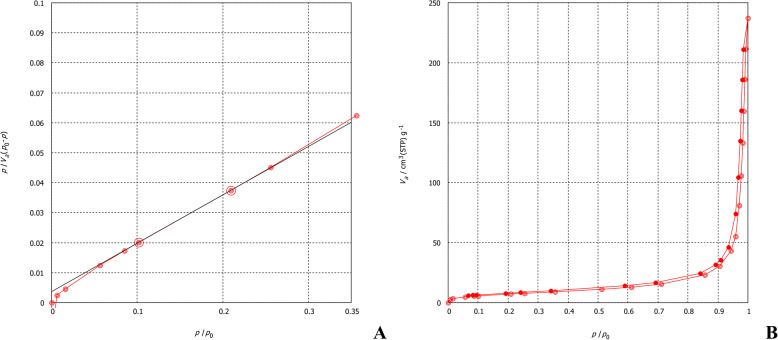
BET Plot (**A**), Adsorption / desorption isotherm (**B**) of FeSNPs nanoparticles

Brunauer-Emmett-Teller (BET) analysis (Fig. 8A, B) provides a precise measurement of the specific surface area (a _S, BET_) of the produced FeSNPs powder, which was found to be 26.371 m^2^/g, with a total pore volume of 0.2916 cm^3^/g. The mean calculated pore diameter was 44.230 nm. The zeta potential of the superparamagnetic iron sulfide nanoparticles was determined to be -16.8 ± 0.6 mV.

### Antagonistic activity

**Fig. 9:**
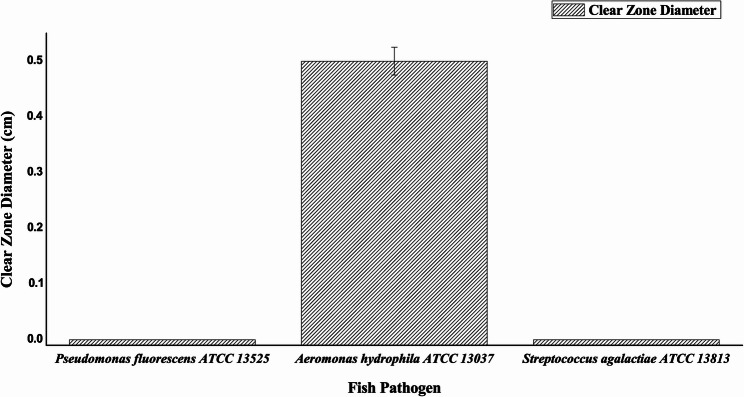
Antagonistic activity of FeSNPs on *Aeromonas hydrophila* (ATCC 13037) fish pathogen

The antagonistic effect of the biosynthesized superparamagnetic iron sulfide nanoparticles (FeSNPs) on some fish pathogens was implemented. Nevertheless, not all iron sulfides exhibit antibacterial activity. In this regard, FeSNPs showed significant antibacterial activity and selectivity toward gram-negative bacteria, particularly *Aeromonas hydrophila* (ATCC 13037), with a clear zone of inhibition of 0.5 cm. The biosynthesized FeSNPs have no antibacterial activity against both fish pathogens, the gram-negative *Pseudomonas fluorescens* Migula 1895^AL^ (ATCC 13525) and the gram-positive *Streptococcus agalactiae* Lehmann and Neumann 1896^AL^ (ATCC 13813) (Fig. 9).

#### MIC and MBC of FeSNPs against pathogenic *Aeromonas hydrophila*

**Fig. 10:**
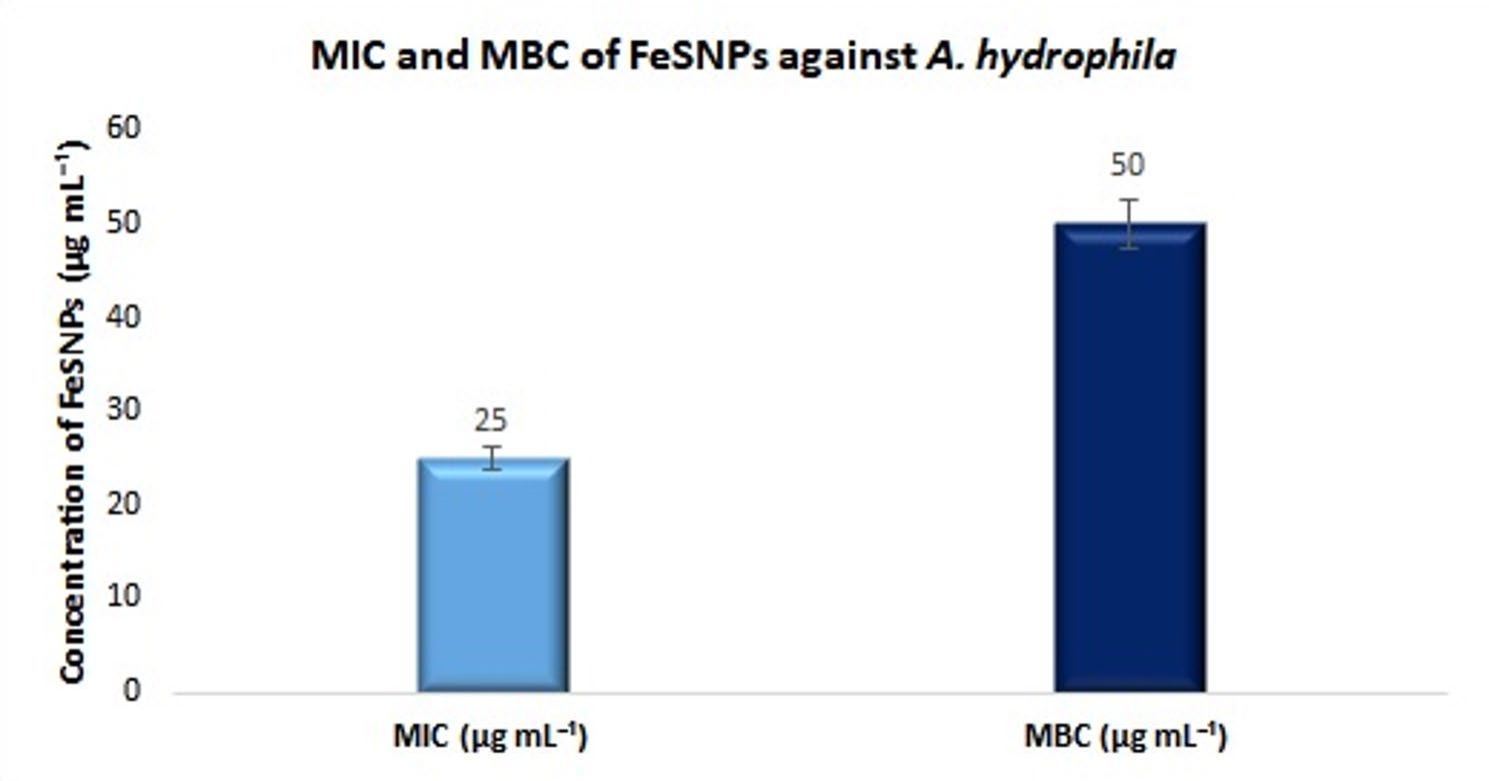
MIC and MBC of FeSNPs against *Aeromonas hydrophila* (ATCC 13037). Data represent mean of three independent experiments

The antibacterial activity of the biosynthesized superparamagnetic iron sulfide nanoparticles (FeSNPs) was quantitatively evaluated against *Aeromonas hydrophila* (ATCC 13037) using the broth microdilution method. FeSNPs exhibited a clear, dose-dependent inhibitory effect on bacterial growth. The minimum inhibitory concentration (MIC), defined as the lowest concentration that completely inhibited visible growth, was determined to be 25 µg/mL. The minimum bactericidal concentration (MBC), defined as the lowest concentration resulting in complete bacterial killing upon subculture, was found to be 50 µg/mL (Fig. 10).

### Cytotoxicity of FeSNPs against cancer and normal cell lines

**Fig. 11:**
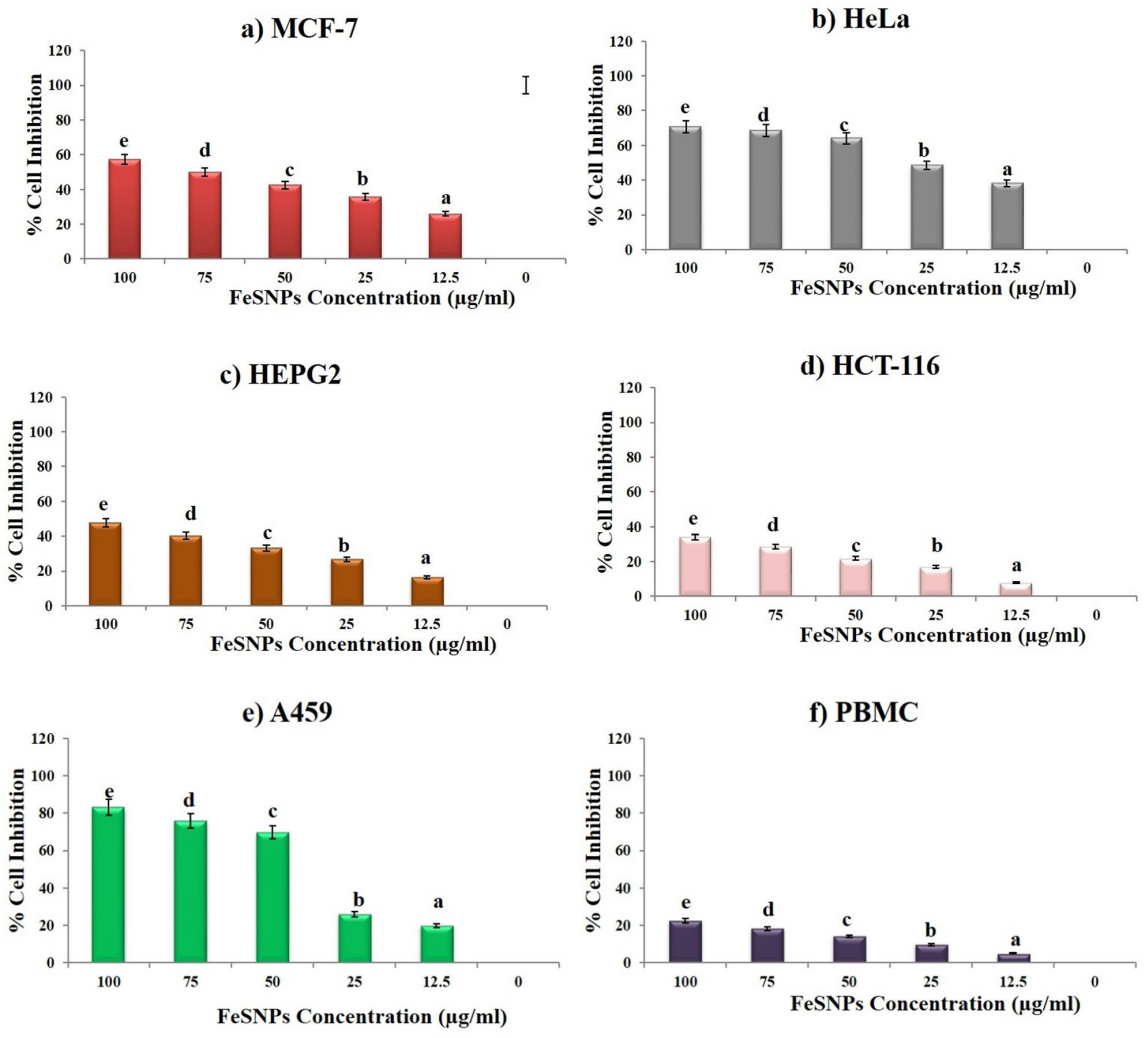
The inhibitory effect of FeSNPs against the different cell lines; (**a)** MCF-7, (**b)** HeLa, (**c)** HEPG2, (**d)** HCT-116, (**e)** A459, and (**f**) PBMC, following 24 h exposure. Data are presented as mean ± SD (*n* = 3 independent biological replicates). Statistical significance was determined using one-way ANOVA followed by Duncan post hoc test (*p* < 0.05)

The cytotoxic activity of FeSNPs against different cancer cell lines and normal PBMCs following 24 h exposure is illustrated in Fig. 11. FeSNPs induced a clear concentration-dependent inhibitory effect across all tested cancer cell lines, including MCF-7, HeLa, HEPG2, HCT-116, and A549. At the highest tested concentration (100 µg/mL), FeSNPs exhibited significant cell growth inhibition, which progressively increased with increasing FeSNPs concentration, indicating dose-responsive anticancer activity. Among the cancer cell lines, A549 cells showed the highest sensitivity to FeSNPs treatment, followed by HeLa and MCF-7 cells, while HEPG2 and HCT-116 cells displayed comparatively moderate inhibition levels. At lower concentrations (12.5–25 µg/mL), a significant but reduced inhibitory effect was still observed in all cancer cell lines, confirming the sustained biological activity of FeSNPs even at minimal doses. In contrast, normal PBMCs demonstrated markedly lower susceptibility to FeSNPs exposure across all tested concentrations, with minimal inhibition observed relative to cancer cells. This differential response highlights the selective cytotoxicity of FeSNPs toward malignant cells while sparing normal immune cells. Statistical analysis confirmed significant differences among concentrations, as indicated by different superscript letters (*p* < 0.05). The IC₅₀ values of FeSNPs demonstrated variable sensitivity among the tested cell lines. A549 cells exhibited the highest sensitivity with an IC₅₀ of 18 µg/mL, followed by HeLa cells (27 µg/mL) and MCF-7 cells (75 µg/mL). In contrast, HEPG2 and HCT-116 cells showed IC₅₀ values exceeding 100 µg/mL, indicating lower susceptibility. Moreover, normal PBMCs also exhibited an IC₅₀ greater than 100 µg/mL, confirming the selective cytotoxicity of FeSNPs toward cancer cells. Overall, these results demonstrate that FeSNPs exert potent and selective anticancer effects in a dose-dependent manner, with limited cytotoxicity toward normal PBMCs.

### Molecular modeling

**Fig. 12:**
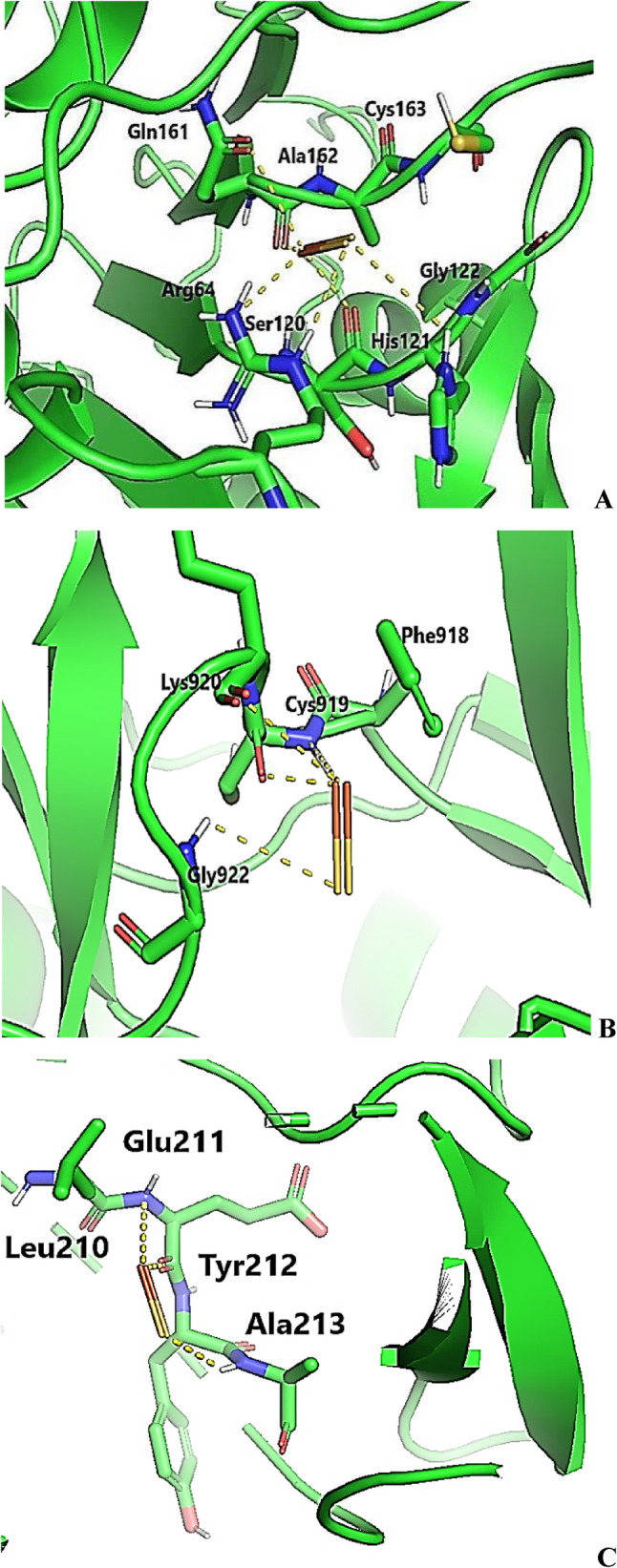
Predicted FeSNPs surface interactions into the ATP-binding sites of Caspase-3 (**A)**, VEGFR-TK (**B)**, and Aurora A (**C**)

Molecular docking simulations were conducted to predict potential binding interactions between FeS nanoparticles (FeSNPs, ~ 5 nm core) and key target proteins (Caspase-3, VEGFR2, Aurora A), serving as hypothesis generation for subsequent biochemical validation. The modeling studies yielded a CDOCKER energy score of -13.6. Figure 12A depicts the binding of FeSNPs to the active site of caspase-3, involving seven key residues: Arg 64, Ser 120, His 121, Gly 122, Gln 161, Ala 162, and Cys 163. The sulfur atom of FeSNPs formed two hydrogen bonds with Ser 120 and His 121, while the iron atom established coordinate bonds with Arg 64, His 121, Gln 161, Ala 162, and Cys 163. Furthermore, the molecular docking studies demonstrated that FeSNPs nanoparticles are optimally positioned at the ATP binding site of VEGFR-2 kinase with a CDOCKER energy score of -11.6. Specifically, FeSNPs occupy the adenine binding pocket and form conserved hydrogen bonds with Cys919, Lys920, and Gly922 in the hinge region (Fig. 12B). An extensive docking study was conducted on Aurora-A to explore its inhibitory activity against lung cancer, hepatocellular carcinoma, cervical squamous cell carcinoma, and breast cancer. The results indicated that FeSNPs nanoparticles exhibit an exceptional fit within the ATP binding site of Aurora-A, with a CDOCKER energy score of -12.6. FeSNPs form hydrogen bonds with the hinge region, specifically with residues Glu211, Tyr212, and Ala213 (Fig. 12C). These key interactions substantially enhance the in vitro potency of FeSNPs.

#### *In vitro* enzyme assays

Induction of active Caspase-3 in A549 cells: To validate the apoptotic potential suggested by docking, the levels of active Caspase-3 were measured in A549 cell culture supernatants following treatment with FeSNPs. The treatment resulted in a 14.6-fold increase in active Caspase-3 levels (516.8 ± 20.1 pg/mL) compared to the control group (34.9 ± 1.37 pg/mL). Inhibition of VEGFR2 and Aurora-A Kinase activity: Experimental assays were conducted to determine the half-maximal inhibitory concentration (IC_50_) of the FeSNPs compared to established inhibitors. VEGFR2 inhibition: FeSNPs exhibited a potent IC_50_ of 0.447 ± 0.019 µg/mL. While less potent than the reference drug Sorafenib (IC_50_ =0.126 ± 0.005 µg/mL), it demonstrated significant anti-angiogenic potential. Aurora-A inhibition: FeSNPs achieved an IC_50_ of 0.268 ± 0.012 µg/mL, compared to 0.051 ± 0.002 µg/mL for the reference inhibitor Danusertib.

## Discussion

The synthesis of metallic nanoparticles has significantly increased in recent decades, but challenges like high costs, energy requirements, and toxic chemicals limit their use. Therefore, bio-fabrication using microorganisms like bacteria, fungi, actinomycetes, and viruses has gained attention as an alternative method. Bacteria are preferred for their diversity and superior growth regulation [61]. For instance, there are several published articles concerning the utilization of *Bacillus* strains for the biological fabrication of metal and metal oxide nanoparticles. *Bacillus* spp. can reduce various metals like gold [62], copper [63], and iron oxides [32, 34] to their nanoscale forms, which are considered a potential bionanofactory for medical applications. The manufacture of iron sulfide nanoparticles (FeSNPs) and their use are significant research areas. Careful production of FeSNPs nanostructures is necessary to modify their properties, enhance their performance, and tailor them to specific applications [64]. These nanoparticles are a unique material for biomedical applications owing to their high surface area and electrocatalytic properties [65]. In our study, we discovered that the thermophilic *Bacillus sonorensis* SS1 (Fig. 1A) can create unique iron sulfide nanoparticles (FeSNPs). The strain was identified, the sequence was uploaded to the GenBank of the NCBI with the accession number SS1 OQ195211, and a phylogenetic tree was created (Fig. 1B). *Bacillus sonorensis* SS1 produced pure black nanopowder (FeSNPs) (Fig. 2A, B, C) that stayed stable at high temperatures (60 °C) and pH levels (pH 9). This means that it could be used in industrial processes that need to work at high temperatures and pH levels. There was no previous research that indicated *Bacillus* species could make iron sulfide nanoparticles. Instead, previous studies have shown a simple way to make size-controlled biogenic iron sulfide nanoparticles (FeSNPs) using *Shewanella*. These FeSNPs were then used to remove Cr(VI) [66]. Another study reported the synthesis of hydrophilic Fe_3_S_4_ nanoparticles was made using a simple hydrothermal technique. This was done by reacting FeSO_4_ with L-cysteine in water at 220 °C for 20 h [10]. Asoufi et al. [67] employed an environmentally friendly approach to create iron sulfide (FeS) nanoparticles by mixing sodium sulfide nonahydrate (Na_2_S.9H_2_O) and ferrous chloride tetrahydrate (FeC_l2_.4H_2_O) with an extract from *Artemisia herba-alba* leaves that acted as a reducing and a stabilizing agent. FeS nanoparticles were produced by Agnihotri et al. [65] using a simple chemical precipitation method, utilizing iron chloride and sodium dithionite as sources for iron and sulfur, and sodium borohydride as a reducing agent. Paca and Ajibade [68] used phenyldithiocarbamate, dimethyldithiocarbamate, and imidazolyldithiocarbamate as single-source precursors to make iron sulfide nanoparticles by heating them in oleic acid/octadecylamine (ODA) at 180 °C. The nanoparticles were mixed with hydroxyethyl cellulose (HEC) to synthesize iron sulfide/HEC nanocomposites.

According to Mathew et al. [64] they described the use of scattered X-rays to look at a substance’s crystal structure, phase composition, and properties. Measurements of atom distances in the crystal lattice help them understand crystalline materials’ composition and properties. Therefore, the X-ray diffraction (XRD) patterns of the produced iron sulfide nanoparticles (FeSNPs) (Fig. 3) show ten peaks with four major reflections at 2θ positions. These peaks correspond to the crystalline phase of iron sulfide with the chemical formulas Fe_0.985_S and Fe_1−x_S. The crystallite sizes of the four main reflections were 17, 17, 18, and 20 nm, with a notable peak at 31.0° at 17 nm. These findings aligned with those previously published by Mathew et al. [64], who demonstrated the lack of peaks belonging to any compounds apart from FeS. We compared the collected peaks with reference JCPDS no. 29–0723 and observed that they matched well. The detected peaks have Miller indices of (111), (200), (100), (210), (211), (220), (311), and (023). Also, Fu et al. [10] showed the X-ray diffraction (XRD) pattern of the magnetic iron sulfide nanoparticles closely aligned with that of greigite-structured Fe_3_S_4_ (JCPDS file no.: 16–0713), demonstrating the successful synthesis of pure greigite-structured Fe_3_S_4_ with high crystallinity. The appropriately generated FeSNPs produced highly dispersible spherical nanoparticles, as seen in Fig. 4’s transmission electron microscope (TEM) image. FeSNPs measured an average size of 4.5 nm, according to the size distribution curve from the TEM image. The size of the FeSNPs produced by *Bacillus sonorensis* SS1 was smaller than the size of the nanocrystals (NCs) Fe_3_S_4_ produced by Fu et al. [10], as confirmed by the transmission electron microscope (TEM) image, which demonstrated that the size was found to be 17.7 nm. A normal TEM picture of freshly prepared FeSNPs by Asoufi et al. [67] clearly showed that the nanostructures were all the same and that the FeSNPs had spherical shapes. The TEM examination depicted nanospheres with an average diameter of 40 nm. Energy dispersive X-ray spectroscope (EDX) is widely used in biomedical research due to its high sensitivity in detecting various elements. The EDX analysis (Fig. 5) shows iron particles constitute 42.25 wt% of the total weight, followed by sulfur particles at 3.68 wt%, oxygen at 37.25%, sodium at 9.24%, carbon at 7.46%, and silver at 0.11%. Paca and Ajibade [68] showed the EDS spectrum of FeS_3_ nanoparticles, which had unique Fe and S peaks. These peaks show that the iron sulfide nanoparticles are present and confirm that they were successfully formed. Mathew et al. [64] performed the EDX analysis of synthesized FeS particles and revealed that they consist solely of iron and sulfur particles, devoid of impurities or other particles. Nickel particles were detected due to the use of nickel foam as a substrate. The iron particles had a weight% of 1.3 wt%, sulfur particles 1.1 wt%, and nickel 7.1 wt%. The EDX analysis confirms the sample’s purity. The FTIR analysis of FeSNPs nanoparticles (Fig. 6) reveals several functional groups that act as surface ligands, improving their biocompatibility. The IR spectrum reveals unique peaks at 3787.6131, 3346.1489, 1628.0517, 1416.6823, 1370.7494, 1107.2268, 897.7119, 572.0903, 445.9431, and 415.1934 cm⁻^1^, corresponding to O–H, N–H, C = C, S = O, N-O, and P–S stretching vibrations. Consistent with our findings, Asoufi et al. [67] reported the FT-IR spectrum of *Artemisia herba-alba*, which revealed a complex structure with several peaks reflecting its complex nature. The broad absorption band at 3383 cm^− 1^ indicates alcohol/phenol –OH, carboxylic acid –OH, and N-H amides, stretching vibration. Absorption bands at 2924 cm^− 1^ and 2850 cm^− 1^ are attributed to CH_3_ and CH_2_ stretching modes. The synthesized FeS nanoparticles show strong bands at 671 –428 cm^− 1^, corresponding to vibrations of elongation and deformation. The physical and chemical properties of the leaves extract prevent nanoparticles aggregation. In addition, the magnetic properties of the biosynthesized FeSNPs nanoparticles were studied (Fig. 7). We observed clear ferromagnetic hysteresis loops up to approximately ± 20 KOe, confirming the ferromagnetic nature of the nanoparticles at room temperature. While the measured saturation magnetization (Ms) value of 30.873 emu/g confirms significant ferromagnetic ordering, it is notably lower than the values reported for biosynthesized iron oxide nanoparticles ~ 51.989 emu/g for γ-Fe_2_O_3_ ‑SPIONs and ~ 53.481 emu/g for Fe_3_O_4_-SPIONs [32, 34]. This reduced Ms is characteristic of biosynthesized iron-based nanoparticles and is likely attributed to several factors inherent to the green synthesis process and nanoscale dimensions [69]. The coercivity (Hci) was relatively low at 94.139 G, and the remanent magnetization (Mr) was 3.5297 emu/g, yielding a remanence ratio (Mr/Ms) of approximately 0.114. This low Hci and Mr/Ms ratio significantly below the theoretical 0.5 value predicted for non-interacting single-domain particles with uniaxial anisotropy [70] suggests the FeSNPs exhibit soft magnetic behavior. This softness likely arises from their small particle size (within the superparamagnetic limit but still showing hysteresis at 300 K) [71]. The combination of moderate saturation magnetization and low coercivity makes these biosynthesized FeSNPs potentially suitable for applications requiring soft magnetic materials responsive to external fields, such as targeted drug delivery or magnetic hyperthermia agents [72], where easy magnetization reversal is advantageous. The study also analyzes the surface area and pore size distributions of FeSNPs produced using N₂ adsorption-desorption isotherms (Fig. 8A, B), revealing a BET surface area of 26.371 m²/g. The Barrett-Joyner-Halenda (BJH) approach was applied to measure the average pore diameter, which was found to be 44.230 nm with a pore volume of 0.2916 cm³/g. Deshpande et al. [73] reported a substantial increase in the surface area of sulfur nanoparticles (177 m²/g) compared to sulfur (48 m²/g) produced in the aqueous phase only, as displayed in BET isotherms.

### The potential antibacterial activity of FeSNPs

The study investigated the antibacterial activity of biosynthesized superparamagnetic iron sulfide nanoparticles (FeSNPs) on some fish pathogens. FeSNPs showed good antibacterial activity against *Aeromonas hydrophila* (ATCC 13037), while they did not show any antibacterial activity against gram-negative *Pseudomonas fluorescens* Migula 1895AL (ATCC 13525) and gram-positive *Streptococcus agalactiae* Lehmann and Neumann 1896AL (ATCC 13813) (Fig. 9). This may be explained by Duan and Sun [9], who stated that Gao’s group developed nano-iron sulfides from garlic, which have shown significant antibacterial activity over 500 times. These sulfides have enzyme-like activity, catalyzing H₂O₂ to generate free radicals and facilitate lipid peroxidation in the cell membrane. They also produce hydrogen sulfide (H_2_S) gas, which can kill bacteria by increasing the amounts of ROS and lipid oxidation, which kills the bacteria. Specifically, Fe_3_S_4_ and Fe_7_S_8_ show significant antibacterial activity and selectivity against gram-negative bacteria. Nano-iron sulfides have special antibacterial properties, potentially classifying them as a class of non-antibiotic drugs that can be used to treat and prevent bacterial vaginitis, cavities in the teeth, and wound infections.

### The potential anticancer activity of FeSNPs on cell lines

Iron sulfide nanoparticles have attracted interest for biomedical applications due to their relatively safe and non-hazardous behavior in biological systems and favorable physicochemical characteristics. In the present study, the biosynthesized FeSNPs exhibited superparamagnetic properties and nearly uniform surfaces and minute particle sizes (4.5 nm), which are known to enhance their biological activity. Moreover, FeSNPs are negatively charged (− 16.8 ± 0.6 mV), a property that may reduce nonspecific interactions with other negatively charged cell membranes, making them less toxic than neutral or positively charged nanoparticles. This negative surface charge is known to promote colloidal stability via electrostatic repulsion, thereby reducing aggregation in biological environments and contributing to improve biocompatibility with extended in vivo half-life [74]. Additionally, biosynthesized iron nanoparticles are typically capped by biologically derived molecules, improving their stability and biocompatibility in cell culture systems [75]. Comparable influences of particle size and surface-associated biomolecules on cytotoxic responses have been reported for iron-based nanoparticles, which were shown to maintain sufficient stability during cytotoxicity assays, leading to reproducible biological effects [76–78]. The cytotoxicity results demonstrated a clear concentration-dependent increase in growth inhibition across all tested cancer cell lines following exposure to FeSNPs at concentrations ranging from 12.5 to 100 µg/mL. Increasing nanoparticle concentration resulted in progressively higher inhibition percentages, indicating effective interaction with cancer cells [45–46]. This dose-dependent cytotoxic response is consistent with previous studies on iron sulfide–based nanocarriers, where higher concentrations led to increased intracellular accumulation and reduced cell viability [9, 79]. Differences in sensitivity toward FeSNPs were evident among the tested cancer cell lines, as reflected by the calculated IC₅₀ values. A549 cells showed the highest sensitivity with an IC₅₀ of 18 µg/mL, followed by HeLa cells with an IC₅₀ of 27 µg/mL. MCF-7 cells exhibited moderate sensitivity with an IC₅₀ of 75 µg/mL, whereas HepG2 and HCT-116 cells were less responsive, with IC₅₀ values exceeding 100 µg/mL. Similar variations in cytotoxic response among different cancer cell lines have been reported for iron sulfide nanocarriers and are commonly attributed to intrinsic biological and metabolic differences [80, 81]. The selectivity of FeSNPs toward cancer cells was further supported by their low cytotoxicity toward normal PBMCs, with an IC₅₀ value exceeding 100 µg/mL and an inhibition percentage of 22.45 ± 0.8. According to ISO 10993-5:2009, materials are considered non-toxic when cell viability exceeds 70%, and the FeSNPs evaluated in this study are classified as non-toxic [82]. Comparable selectivity patterns, where green synthesized iron sulfide–based nanomaterials exert stronger effects on cancer cells than on normal cells, have been reported previously and support the biocompatible nature of these nanostructures [83–86]. Overall, the present findings indicate that biosynthesized FeSNPs induce concentration-dependent and cell line–specific cytotoxic effects, with significant activity against A549 and HeLa cells and minimal effects on normal PBMCs. The obtained IC₅₀ values, together with the favorable physicochemical characteristics of the nanoparticles, are consistent with previously reported iron sulfide–based systems and highlight the potential of FeSNPs for further investigation as anticancer agents [9, 79, 80].

### Molecular modeling

It is well established that caspase-3, a cysteine protease, plays a crucial role in the apoptotic cell death of lung, breast, and liver tumor cells [87–90]. To simulate the interactions between the synthesized nanoparticles and target proteins, the computational approach was adapted from classical small-molecule docking to nanoparticle surface-interaction modeling. The FeSNP model was constructed based on experimentally determined parameters, including a 4.5 nm average core size (TEM) and a hexagonal crystalline structure confirmed by XRD analysis. FeSNPs the “ligand” in this study represented a representative surface fragment of the FeS lattice. This fragment incorporated the surface functionalities identified via FTIR, such as O–H, N–H, and S = O groups, which contribute to the observed negative surface charge of − 16.8 ± 0.6 mV.

To account for the size disparity between the nanoparticle and the protein active sites, docking was performed using an enlarged grid (30 × 30 × 30 Å). This allowed for the simulation of multi-point surface binding rather than rigid occupancy. The model specifically evaluated how the surface-exposed Iron (Fe) atoms established coordinate bonds and Sulfur (S) atoms formed hydrogen bonds with key residues in Caspase-3, VEGFR2, and Aurora-A. This surface-mediated approach provided a mechanistic hypothesis for the subsequent biochemical validation, where FeSNPs demonstrated potent enzyme inhibition and a 14.6-fold induction of active Caspase-3. The strong binding interactions of FeSNPs with caspase-3 suggest that they may enhance apoptosis in cancer cells by directly activating this pathway. The observed hydrogen and coordinate bonding interactions reinforce the potential of FeSNPs as effective caspase-3 activators. In relation to VEGFR-TK, it is noteworthy that VEGF is a pivotal angiogenic factor in tumors, significantly contributing to the early stages of tumor development, progression, and metastasis. As a result, VEGF and its receptor-mediated signaling pathways have emerged as critical therapeutic targets in the treatment of various cancers. Presently, anti-VEGF-based antiangiogenic drugs (AADs) are extensively utilized in clinical practice for managing diverse cancer types in human patients [91–93]. The molecular docking results indicate that FeSNPs may interfere with VEGFR-TK activity, thereby reducing cellular proliferation and tumor growth. Regarding Aurora-A, overexpression of this kinase induces resistance to radiotherapy in various cancers, including lung cancer, hepatocellular carcinoma, cervical squamous cell carcinoma, glioblastoma, nasopharyngeal carcinoma, breast cancer, and prostate cancer [94–97]. The strong binding interactions observed in the docking study suggest that FeSNPs may serve as effective inhibitors of Aurora-A, potentially overcoming resistance mechanisms in radiotherapy. These findings highlight the potential therapeutic relevance of FeSNPs in targeting Aurora-A and other oncogenic pathways. It’s worth noting that the classical docking algorithms optimized for small molecules (< 500 Da) have inherent limitations when modeling nanoparticles. Consequently, future studies will be conducted to employ enhanced sampling MD simulations and machine learning-based affinity predictions to better characterize dynamic FeSNPs-protein surface interactions observed in the current biochemical validation.

#### Mechanistic insights into the synergistic activation of apoptosis and inhibition of proliferative Kinases by FeSNPs

The biological results corroborate the molecular modeling study, suggesting that FeSNPs serve as multifunctional inhibitors of tumor growth and angiogenesis. The results of the surface-interaction modeling, which predicted coordinate bonding with the Caspase-3 catalytic residue Cys163, were experimentally validated by a dramatic 14.6-fold increase in active Caspase-3 levels. This high level of enzymatic activation (516.8 ± 20.1 pg/mL) confirms that the multi-point surface binding of FeSNPs effectively triggers the programmed cell death pathway in lung cancer (A549) cells.

The potency of FeSNPs against proliferative kinases was further evidenced by sub-microgram IC_50_ values, which closely aligned with the high CDOCKER scores obtained in the ATP-binding site simulations. Specifically, the IC_50_ of 0.447 ± 0.019 µg/mL for VEGFR2 supports the model’s prediction that the FeS lattice surface obstructs the hinge region, potentially disrupting tumor metastasis and vascularization. Similarly, the potent Aurora-A inhibition (IC_50_ = 0.268 ± 0.012 µg/mL) validates the affinity of FeSNPs for this kinase, suggesting a promising mechanism for overcoming radiotherapy resistance in various malignancies. Collectively, these findings highlight the therapeutic relevance of FeSNPs. Their dual-action mechanism simultaneously activating apoptosis via Caspase-3 and inhibiting proliferative kinases like VEGFR and Aurora-A positions them as promising candidates for advanced cancer therapy.

This work shows that it is possible to make FeSNPs using microbes; however, there are some constraints that need to be taken into account to understand the results. The choice of strain was based on the screening procedure of all isolates that confirmed only this strain successfully produced FeSNPs, which were identified molecularly as *Bacillus sonorensis*. There was very limited information about this strain in previous research and no data on iron reduction; additionally, it remains unclear how using different types of microbes will affect the attributes of nanoparticles, such as their size distribution and crystallinity. Considering that the biological synthesis of magnetic nanoparticles allows many functional groups on the surface that function as a surface coating, shape controllers, and stabilizing agents, which was confirmed by the FTIR analysis performed during this study. These functional groups facilitate the bonding with the nanoparticles. This concept was confirmed by Yuan et al. [17], who cited some previous studies that have examined the stability of FeS₂ in different temperatures and the concentrations of absorbed water on the surface. Temperature has little effect on the morphology of FeS₂ under low absorbed water concentrations. However, above 90 K, the conversion from an octahedral structure to a cubic shape is promoted. At higher concentrations of water, the dependence on temperature is more apparent. Latter study established that the functions of the surface ligands affect the stability of FeS₂ (FeS₂ nanorods synthesized in the laboratory). The stability of FeS contributed to low energy excitation from Fe d to S-Sσ*p. Fe₃S₄ was observed at 200 °C for 30 h, then it transformed to FeS₂ over time. The current study did not test how stable the nanoparticles would be in the long term in terms of colloids and chemicals in the environment, as well as the polydispersity of the nanoparticles, which is important for their practical use. The synthesis was done on a small scale, so scaling up may lead to reduced reproducibility due to mixing differences. Finally, the in vitro confirmation of antibacterial activity, although promising, is preliminary; the mechanisms of action and efficacy are complicated. Subsequent study will concentrate on addressing these problems.

## Conclusion

The thermophilic *Bacillus sonorensis* SS1, isolated from marine sediments, serves as an efficient and eco-friendly bio-factory for the synthesis of superparamagnetic iron sulfide nanoparticles (FeSNPs). These nanoparticles possess unique physicochemical properties, including an average particle size of 4.5 nm, a negative surface charge (− 16.8 ± 0.6 mV), and exceptional stability at high temperatures (60 °C) and alkaline pH (pH 9). The presence of biogenic capping groups, such as O–H and N–H, identified via FTIR, acts as surface ligands that enhance the biocompatibility and colloidal stability of the particles. The molecular docking studies of FeSNPs against Caspase-3, VEGFR, and Aurora-A may provide compelling insights into their potential as therapeutic agents for cancer treatment. The findings revealed that FeSNPs potentially bind to the active sites of these key proteins, indicating their possible activity to inhibit critical pathways involved in tumor development and progression. The integration of nanoparticle surface-interaction modeling and biochemical validation provides a comprehensive understanding of the therapeutic potential of biosynthesized FeSNPs from *Bacillus sonorensis* SS1, demonstrating their ability to coordinate with active sites of Caspase-3, VEGFR2, and Aurora-A to inhibit critical oncogenic pathways. Computational modeling predicted crucial coordinate bonds with Cys163 of Caspase-3, experimentally confirmed by a dramatic 14.6-fold increase in active enzyme levels (516.8 ± 20.1 pg/mL) in A549 cells, establishing FeSNPs as potent apoptosis inducers. Similarly, surface-mediated binding to proliferative kinases translated into highly potent inhibition, with FeSNPs occupying the VEGFR2 ATP-binding pocket (IC_50_ = 0.447 ± 0.019 µg/mL) to disrupt angiogenesis and tumor metastasis, and exhibiting high affinity for the Aurora-A hinge region (IC_50_ = 0.268 ± 0.012 µg/mL) to counteract radiotherapy resistance. Collectively, this dual-action mechanism simultaneously triggers apoptosis while blocking tumor signaling combined with selective cytotoxicity (A549 IC_50_*=* 18 µg/mL; normal PBMCs IC_50_ >100 µg/mL), positioning FeSNPs as safe, multifunctional candidates for advanced cancer therapy, though in vivo studies are needed to evaluate pharmacokinetics and clinical translation.

## Data Availability

The investigation’s original findings were presented in the article. The 16S rRNA sequence of *Bacillus sonorensis* SS1was submitted to the National Center for Biotechnology Information (NCBI) GenBank under the accession number SS1 OQ195211 (https://www.ncbi.nlm.nih.gov/nuccore/2421924889). The corresponding author can be contacted with any additional questions.
